# Cardiovascular and autonomic nervous system response to graded exercise in adolescents with type 1 diabetes

**DOI:** 10.3389/fendo.2026.1813865

**Published:** 2026-04-22

**Authors:** Tadej Rondaij, Jakob Jesih, Klemen Dovč, Tadej Battelino, Nejka Potočnik

**Affiliations:** 1 Faculty of Medicine, Institute of Physiology, University of Ljubljana, Ljubljana, Slovenia; 2 Faculty of Health Sciences, University of Ljubljana, Ljubljana, Slovenia; 3 Department of Pediatric Endocrinology, Diabetes and Metabolism, University Children’s Hospital, University Medical Centre Ljubljana, Ljubljana, Slovenia; 4 Faculty of Medicine, University of Ljubljana, Ljubljana, Slovenia

**Keywords:** autonomic nervous system, cardiovascular system, exercise testing, microcirculation, oxygen uptake, type 1 diabetes

## Abstract

**Introduction:**

Type 1 diabetes (T1D) is associated with an increased risk of cardiovascular and autonomic complications. Although cardiopulmonary exercise testing (CPET) is a valuable tool for assessing cardiorespiratory function, data on physiological response to maximal exertion in adolescents with T1D remain limited and inconsistent. This study aimed to compare cardiovascular, respiratory, metabolic, and microvascular responses to CPET in adolescents with T1D and healthy peers.

**Methods:**

Sixteen participants aged 11–16 years (eight with T1D and eight healthy controls), matched for anthropometric characteristics, underwent CPET on a cycle ergometer. Respiratory gas exchange, heart rate, heart rate variability, blood pressure, blood glucose, lactate concentration, skin blood flow, skin temperature, and cutaneous vascular conductance were measured at predefined time points during rest, exercise, and recovery. Blood glucose, lactate concentration, and skin microvascular variables were assessed at rest and during recovery.

**Results:**

Adolescents with T1D demonstrated a significantly lower V̇O_2_/power output slope and a higher ventilatory equivalent for oxygen at maximal effort, suggesting altered oxygen uptake efficiency. Maximal power output and maximal oxygen consumption did not differ between groups. Heart rate responses and heart rate variability were similar throughout testing. However, finger skin blood flow and cutaneous vascular conductance were significantly lower in the T1D group at rest and during recovery.

**Conclusion:**

Adolescents with T1D showed preserved cardiovascular function and comparable overall exercise capacity to healthy peers, despite subtle impairments in oxygen utilization and reduced skin microvascular function. These findings indicate that even at a young age, T1D is associated with altered metabolic, respiratory, and microvascular responses to maximal exercise. The results suggest that peripheral, rather than central mechanisms may underlie these differences, potentially involving glucose levels or synthetic insulin effects on vascular endothelium.

## Introduction

Type 1 diabetes (T1D) is characterized by early immune-mediated destruction of insulin-producing beta-cells of the pancreas. The onset of T1D usually occurs in childhood, with reported peak incidence of diagnosis in adolescents aged 10–14 years (1). The global prevalence of T1D is estimated to be 9.5 million people (2). Lifelong insulin replacement therapy is needed and tight glycemic control is paramount to postpone or prevent complications, since glycemic outcomes are closely associated with a higher mortality rate (3).

Chronic hyperglycemia contributes in due course to a range of metabolic complications, such as cardiovascular disease, retinopathy, nephropathy, and peripheral neuropathy. These complications usually correlate with the duration of diabetes; however, evidence suggests that adolescents with T1D may already exhibit early signs of vascular dysfunction and atherosclerosis (4).

As a result of chronic elevated blood glucose levels, the autonomic nervous system in individuals with T1D may be exposed to metabolic stress, inadequate blood supply and autoimmune damage, which may lead to the development of diabetic autonomic neuropathy (5), a common and serious complication of diabetes. Signs of autonomic impairment in individuals with T1D may present as soon as within 2 years following diagnosis, sometimes years preceding manifestation of clinical symptoms (6). This type of neuropathy may affect any part of the autonomic nervous system, potentially impairing multiple organ systems, leading to increased morbidity and mortality. Most commonly, it involves impairment of the cardiovascular, gastrointestinal and genitourinary systems, as well as metabolic, pupillary and sudomotor dysfunction. Cardiac autonomic neuropathy (CAN) is perhaps the most clinically significant form of autonomic impairment in diabetic individuals, which has been shown to be associated with an increased risk of silent myocardial ischaemia, cardiac arrhythmias, as well as sudden death (7, 8). The prevalence of CAN in individuals with newly diagnosed T1D is estimated to be around 7%, with an annual increase of approximately 2% (9). The earliest clinical indicator of cardiac autonomic impairment is thought to be reduced heart rate variability (HRV), a function of the interplay of sympathetic and parasympathetic activity, which regulates the cardiac response to the body’s level of metabolic activity and changes in blood flow (7). Reduced HRV may already be present in adolescents with T1D (10, 11), and those affected exhibit reduced work capacity at set heart rates compared to their peers with T1D without autonomic dysfunction (12).

Metabolic and cardiovascular responses to exercise in individuals with T1D have mostly been studied on the adult population (13, 14). Several studies reported decreased maximal oxygen uptake in individuals with T1D (14–16), which may be related to altered O2 transport and tissue delivery during exercise (16). These observations support the rationale for examining oxygen kinetics in response to exercise in adolescents with T1D.

Physical activity seems to exert a generally positive impact on lipid profiles and endothelial function in diabetic children and adolescents, and is associated with reduced early signs of atherosclerosis (17). While conflicting data exists on the effect of exercise training on glycemic outcomes (18, 19), a recent meta-analysis suggested a moderate positive effect of this intervention on glycated haemoglobin levels in children and adolescents with T1D (20). Similarly, evidence from studies on the impact of T1D on maximal exercise capacity and physiological responses to exercise in children and adolescents with T1D remains inconclusive (21, 22).

In individuals with diabetes, structural and functional alterations occur in the microvasculature, which may affect the capillary basement membrane in tissues such as the glomeruli, retina, myocardium, skin, and skeletal muscle (23). Impaired skin microcirculation response to various local physical and pharmacological stimuli in children and adolescents with T1D has been well documented (24, 25). Additionally, exercise training has been shown to potentially delay or attenuate endothelial dysfunction in both young and adult individuals with diabetes (26, 27), while greater levels of aerobic fitness may be associated with improved vasodilatory capacity of the skin microcirculation (28). However, limited data exists regarding the effect of T1D on cutaneous microvascular response to dynamic exercise in diabetic adolescents compared to their heathy peers.

Cardiopulmonary exercise testing (CPET) can be used to accurately assess physiological responses to short-term maximal exercise and is commonly performed in sports medicine (29). CPET provides the most accurate noninvasive quantification of maximal aerobic capacity and participant effort and is safe to perform on individuals with T1D, provided adequate safety measures are implemented (30). The aim of this study was to determine whether adolescents with T1D exhibit differences in metabolic, respiratory, cardiovascular, cardiac autonomic and skin microvascular responses to short-term dynamic exercise to exhaustion, compared to healthy peers, using CPET.

We hypothesized that, compared with healthy peers, adolescents with T1D would exhibit an altered physiological response to maximal graded exercise, characterized by impaired cardiac autonomic modulation, altered oxygen kinetics, and reduced skin microvascular reactivity. The primary study endpoints were between-group differences in parameters of cardiorespiratory efficiency, skeletal muscle metabolism, autonomic cardiovascular function, and peripheral microvascular function.

## Materials and methods

In this prospective observational study, we included adolescents aged 11–16 years with T1D, as well as healthy peers, included by convenience sampling. Healthy participants were matched to the experimental group based on sex, age and body mass index (BMI). Exclusion criteria comprised any condition other than T1D, including the presence of neurological or musculoskeletal disorders that could interfere with participation in the study. Habitual physical activity was assessed using the International Physical Activity Questionnaire (IPAQ), and the total physical activity score (MET-minutes/week) was used to characterize activity levels of participants. Sexual maturity of the study participants was assesed using the Tanner score. Two female subjects in each group had already reached menarche, and measurement were taken during the early follicular phase of the menstrual cycle to minimize hormonal effects on physiological parameters. At the time of the study, all adolescents with T1D were using continuous glucose monitoring and insulin pump in an open loop configuration. ﻿Glycemic strategy was managed as per international guidelines by Moser et al. (31). CPET was initiated only when plasma glucose was ≥ 5.0 mmol/L (90 mg/dL), with no changes to insulin dosing.

Informed consent was obtained from all participants’ parents or legal guardians, and the study was approved by the National ethics committee of the Ministry of health. Participant information was anonymized.

Measurements were conducted in the Exercise testing laboratory at the Institute of Physiology, Faculty of Medicine, University of Ljubljana. To ensure participant safety, medical personnel were on standby throughout the testing procedures. CPET was conducted in the morning. Participants were asked to consume a meal at least one hour before testing, and participants with T1D were instructed to maintain their usual insulin regimen. Upon arrival at the laboratory, where the room temperature was controlled at 21 °C, participants’ anthropometric measurements were recorded. All physiological parameters, except for blood glucose and lactate concentrations, were monitored using non-invasive methods. Blood pressure before testing was measured with an automated sphygmomanometer to confirm normotension, while continuous monitoring during the exercise testing and recovery phase was performed using a finger cuff placed on the right index finger (Finometer, FMS, Netherlands). Blood lactate and glucose concentrations were measured from capillary blood samples obtained from the fingertip prior to and following CPET. Blood lactate concentration was measured using the Lactate Plus analyzer, and blood glucose concentration was determined using the Accu-Check system. Respiratory parameters and O_2_/CO_2_ kinetics were recorded by the Quark system (COSMED, Italy) using a breath by breath method. Cardiac activity was monitored via electrocardiography (ECG) using the standard limb lead II configuration (Finometer, FMS, Netherlands). Changes in microcirculation were assessed by measuring skin temperature and skin blood flow by laser Doppler flowmetry in the forearm skin and finger pulp using the PeriFlux 6000 system (Perimed). Microcirculation was evaluated at rest and in the recovery phase to avoid motion artefacts, as laser Doppler flowmetry measurements are highly sensitive to movement and mechanical vibration during exercise testing. Accordingly, cutaneous vascular conductance (CVC) was calculated as skin blood flow divided by mean arterial pressure in these two time periods. No user-defined filtering or automated artefact-rejection algorithms were applied beyond the manufacturer’s default processing. These variables, ECG and finger pressures were recorded continuously at a sampling rate of 500 Hz using the WinDataQ data acquisition system (DataQ Instruments, USA). All parameters were measured using validated methods for cardiopulmonary exercise testing, hemodynamic monitoring, and microcirculatory assessment.

The CPET was conducted on a cycle ergometer according to the following protocol:

1. 5 minutes of rest (resting phase), during which time baseline measurements were obtained, as well as measurement of blood lactate and blood glucose concentrations.
2. The active phase of the test consisted of graded exercise using a ramp protocol, with workload increasing uniformly by 15 to 30 W per minute, depending on the participant’s anthropometric characteristics and expected exercise capacity (**Table 1**), until one of the following predefined termination criteria was reached: volitional exhaustion indicated by inability to maintain cadence; a plateau in V̇O_2_ or heart rate (HR); RQ over 1.2 in at least three consecutive recorded breath by breath data points, or the participant’s request to stop.
3. Immediately following exercise cessation, blood lactate and glucose concentrations were again determined using a capillary blood sample.
4. The test was concluded with a 10-minute recovery phase, during which time participants remained still and seated on the cycle ergometer and physiological parameters continued to be recorded.

**Table 1:** Anthropometric data, CPET workload protocols and performance outcomes for each study participant.

N	Group	Age [years]	Sex	T	Height [cm]	Weight [kg]	BMI [kg/m^2^]	IPAQ [METmin/week]	Duration of T1D [Years]	HBA1c [%]	POmax [W]	%HRmax [%]
1	T1D	14.7	F	3	165	53	19	968	12.2	7.7	167	91.22
2	T1D	11.9	F	2	146	42	20	855	9.2	7.3	122	89.90
3	T1D	12.4	F	2	151	32	14	1530	5.8	6.6	111	90.87
4	T1D	15.3	M	3	180	70	22	1010	10.0	9.4	266	90.24
5	T1D	14.9	M	3	172	56	19	1259	4.3	7.6	259	93.17
6	T1D	15.8	M	3	178	61	19	1210	4.3	7.0	280	91.67
7	T1D	14.5	M	2	170	59	20	1210	1.4	6.4	270	89.76
8	T1D	15.9	F	4	169	58	20	1213	5.3	7.3	185	90.69
9	Healthy	12.1	F	2	157	43	17	1373			138	87.50
10	Healthy	13.9	M	2	165	50	18	1311			254	85., 92
11	Healthy	15.7	M	3	168	61	22	1210			301	96.57
12	Healthy	15.8	M	3	202	90	22	1383			280	90.69
13	Healthy	15.4	F	4	167	42	15	1050			174	94.61
14	Healthy	15.1	M	2	180	66	20	1210			260	82.93
15	Healthy	12.5	F	2	163	55	21	968			133	89.42
16	Healthy	14.1	F	3	160	45	18	1133			132	89.81
Mean (SD)	T1D	14.42 (1.51)		2.75 (0.71)	166.38 (12.11)	53.88 (11.83)	19.21 (2.26)	1156.88 (209.36)	6.56 (3.57)	7.41 (0.92)	207.50 (69.70)	90.94 (1.11)
Mean (SD)	Healthy	14.33(1.43)		2.625 (0.74)	170.25 (14.54)	56.50 (16.04)	19.15 (2.42)	1204.75 (149.56)			209.00 (71.82)	90.06 (3.86)
p-value		0.96		0.74	0.57	0.72	0.96	0.94			0.74	0.81

T, Tanner score; BMI, body mass index; IPAQ, international score; POmax, maximal work load; %HRmax, percentage of maximal predicted heart rate reached at the end of exercise; M, male; F, female, p value was obtained by t-test for independent samples.

**Figure 1:**
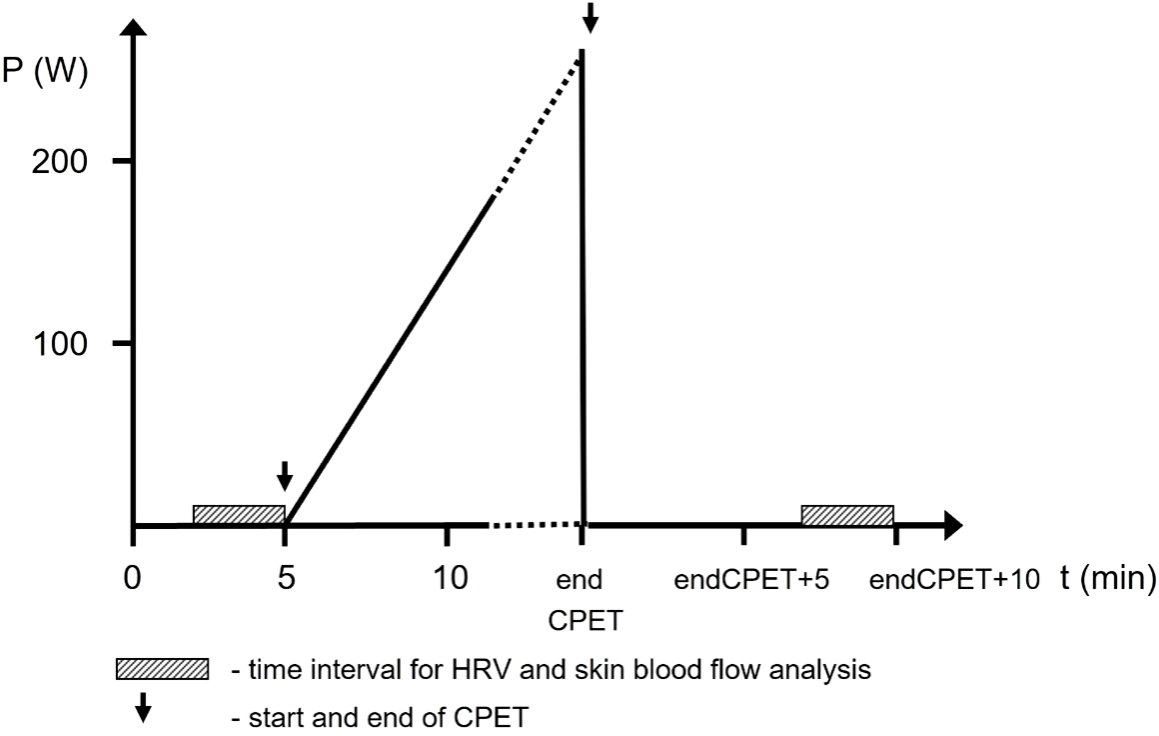
Schematic representation of the CPET protocol.

The parameters measured during the examination and their corresponding time points used in analysis are presented in **Table 2**. A schematic depiction of the measurement protocol is presented in **Figure 1**.

**Table 2:** Parameters measured during the study and their corresponding measurement time points used in analysis.

Physiological system	Abbreviation	Parameter	Measurement time points used in analysis
Musculoskeletal system	PO	Power output	Throughout the active phase
	PO_max_	Maximum power output	Point of highest power output during active phase
Metabolic system	V̇O_2_	Volume of O_2_ consumed per minute	Five time points^†^
	V̇O_2_/PO	Oxygen consumption per power output	Calculated over the active phase time period
	V̇O_2_/kg	Volume of O_2_ consumed per minute per kilogram body weight	Five time points^†^
	V̇O_2max_	Peak oxygen consumption in mL per minute	Point of highest oxygen consumption during active phase
	OUES	Oxygen uptake efficiency slope	Calculated over the active phase time period
	V̇CO_2_	Volume of CO_2_ produced per minute	Five time points^†^
	RQ	Respiratory quotient	Five time points^†^
	GET	Gas exchange threshold	Single time point during active phase
	RCP	Respiratory compensation point	Single time point during active phase
	MET	Metabolic equivalent of task	Five time points^†^
	GC	Blood glucose concentration	CPET -10 min and at PO_max_
	LC	Blood lactate concentration	CPET -10 min and at PO_max_
	T_arm_	Skin temperature in forearm	CPET -1 minute and end CPET
	T_fin_	Skin temperature in finger	From CPET -3 min to start CPET; From CPET + 7 min to CPET + 10 min
Cardiovascular system	HR	Heart rate	Five time points^†^
	RR_rest_	RR interval duration at rest	From CPET -3 min to start CPET
	RR_rec_	RR interval duration in recovery phase	From CPET + 7 min to CPET + 10 min
	HRV	Heart rate variability	From CPET -3 min to start CPET; From CPET + 7 min to CPET + 10 min
	SDNN	Standard Deviation of Normal-to-Normal Intervals	From CPET -3 min to start CPET; From CPET + 7 min to CPET + 10 min
	RMSSD	Root Mean Square of Successive Differences	From CPET -3 min to start CPET; From CPET + 7 min to CPET + 10 min
	LF	Low frequency component of HRV	From CPET -3 min to start CPET; From CPET + 7 min to CPET + 10 min
	HF	High frequency component of HRV	From CPET -3 min to start CPET; From CPET + 7 min to CPET + 10 min
	LF/HF	Ratio between low and high frequency HRV components	From CPET -3 min to start CPET; From CPET + 7 min to CPET + 10 min
	HRR30	Heart rate recovery in 30s	The fall in HR in 30 seconds after end of active phase
	HRR60	Heart rate recovery in 60s	The fall in HR in 60 seconds after end of active phase
	MAP	Mean arterial pressure	Five time points^†^
	BF_arm_	Laser Doppler skin blood flow in the forearm	From CPET -3 min to start CPET; From CPET + 7 min to CPET + 10 min
	BF_fin_	Laser Doppler skin blood flow in the finger pulp	From CPET -3 min to start CPET; From CPET + 7 min to CPET + 10 min
	CVC_arm_	Cutaneous vascular conductance in forearm	From CPET -3 min to start CPET; From CPET + 7 min to CPET + 10 min
	CVC_fin_	Cutaneous vascular conductance in finger	From CPET -3 min to start CPET; From CPET + 7 min to CPET + 10 min
	V̇O_2_/HR	Oxygen pulse	Five time points^†^
Respiratory system	VE	Ventilation	Five time points^†^
	VE/V̇CO_2_	Ventilatory equivalent for CO2	Five time points^†^
	VE/V̇O_2_	Ventilatory equivalent for O2	Five time points^†^
Gas exchange	VE/V̇CO_2_slope	Slope of VE per VCO2	Calculated from rest to GET
	PetCO_2_	End tidal partial pressure of CO2	Five time points^†^
	PetO_2_	End tidal partial pressure of O2	Five time points^†^

† Five measurement time points included the end of the resting phase (REST), the gas exchange threshold (GET), the respiratory compensation point (RCP), the point of maximum power output (PO_max_) and the end of measurement (REC).

Data were imported in MS Excel 2013 and statistically processed using R (R Core Team, 2022) within the RStudio environment (RStudio Team, 2022). The gas exchange threshold (GET) was identified via the V-slope method as the breakpoint in the V̇CO_2_–V̇O_2_ relationship, where V̇CO_2_ began to rise disproportionately due to lactic acid buffering. The respiratory compensation point (RCP) was determined from the VE/V̇CO_2_ plot as the point where ventilation increased disproportionately to V̇CO_2_, reflecting the onset of metabolic acidosis from ongoing blood lactate accumulation (32).

Both thresholds were computed using R code and subsequently marked in the Quark software (Cosmed, Italy), which automatically generated the corresponding values at GET and RCP timepoints. Values of measured parameters at maximal effort (PO_max_) were determined by the Quark algorithm and manually checked for accuracy. For the resting period (REST), the final three minutes prior to the onset of active phase were analyzed, while for the recovery period (REC), the final three minutes of recording (7–10 minutes post-exercise) were selected. These two time intervals were also used for calculating HR, HRV, and MAP in the BPV Analysis software (Nevrokard, Slovenia). Average values for BF and temperature in the finger and forearm at rest and in the recovery were determined in the same time intervals using the WinDataQ software.

The cardiovascular response was assessed by measuring RR interval duration and heart rate variability (HRV) at rest and during recovery, as well as parameters of heart rate decrease during recovery. RR intervals were extracted from the ECG recordings, which automatically identified R-peaks; in cases where the program failed to detect them, manual correction was applied. HRV and MAP were calculated automatically by the software and evaluated as time domain parameters (standard deviation of normal-to-normal intervals (SDNN) and root mean square of successive differences (RMSSD)) and as frequency domain parameters determined by autoregressive method (the power of high-frequency band (HF; 0.15–0.40 Hz), the power of low-frequency band (LF, 0.04–0.15 Hz) and their ration LF/HF) (33). Participants were breathing spontaneously, and the breathing frequency was confirmed to be within the HF band.

Heart rate recovery at 30 and 60 seconds (HRR30, HRR60) was determined by measuring the reduction in HR at 30 and 60 seconds post exercise, respectively.

Using the G*Power for Windows (vs. 3.1.9.7) for F-test, ANOVA repeated measures, within-between interaction with alpha level at 0.05, power at 0.80, 2 groups (Group-2 X Time-2 design), lower bound of sphericity at 1, and effect size at 0.4, yielded a total sample size of 16 (34).

Statistical analysis was performed using SPSS software, version 29 (IBM, New York, USA). For parameters determined only once per participant (e.g., anthropometric characteristics, VE/V̇CO_2_ slope, OUES, V̇O_2_/PO, HRR30 and HRR60), comparisons between groups were made using a t-test for independent samples, where t-test statistics (t), degrees of freedom (df), p value and effect sizes (Cohen’s d), representing the difference between group means in standard deviation units, were reported. For parameters assessed at multiple time points, a two-way repeated-measures ANOVA was used to evaluate the main effects of time and group, as well as their interaction. When a significant omnibus effect was detected, *post hoc* pairwise comparisons were performed. To control for multiple comparisons across time points, Bonferroni correction was applied to the *post hoc* analyses.

For ANOVA analyses, we reported the test statistic (F), p-values, and the proportion of explained variance (partial eta squared, η²). Statistically significant differences were indicated in the figures and tables by *.

## Results

A total of 16 participants were included in this study, of which 8 had T1D (mean duration of T1D 6.6 (SD 3.6) years, HbA1c 7.4% (SD 0.9)), and 8 were healthy peers. The mean age of adolescents with T1D and their healthy peers included in the study was 14.42 and 14.43 years, respectively. Participants’ characteristics with corresponding CPET workload protocol and performance outcomes, as well as differences between groups, are shown in **Table 1**.

The two groups were comparable in anthropometric characteristics and age- and sex- matched. The duration of exertion did not differ significantly between groups and was within a narrow range (8–12 minutes), indicating that participants’ performance capacity was accurately estimated. There was no statistically significant difference between the groups in terms of peak power achieved. At maximal exercise, participants in both groups reached more than 85% of their predicted maximal heart rate, indicating that they achieved their maximal aerobic capacity (35). Mean values of metabolic and respiratory parameters of both study groups calculated for five different time points during CPET are presented in **Table 3**.

**Table 3:** Mean values (standard deviation) of metabolic and respiratory system parameters measured during five time points: the resting phase (REST), the gas exchange threshold (GET), the respiratory compensation point (RCP), the point of maximum power output (PO_max_) and the recovery phase (REC).

Parameter	REST		GET		RCP		PO_max_		REC	
	Healthy	T1D	Healthy	T1D	Healthy	T1D	Healthy	T1D	Healthy	T1D
HR [min^-1^]	95.12 (16.54)	93.25 (13.51)	129.75 (14.17)	122.25 (13.81)	177.18 (15.75)	168.25 (18.56)	184.13 (8.46)	187.38 (2.26)	113.62 (10.36)	111.50 (11.66)
V̇O_2_ [ml]	373.25 (107.43)	372.00 (85.39)	1242.25 (491.45)	1023.63 (321.24)	2019.75 (749.82)	1774.75 (577.71)	2592.13 (929.90)	2150.63 (556.50)	441.38 (139.29)	453.13 (60.08)
V̇O_2_ [ml/kg]	6.64 (0.95)	7.01 (1.20)	21.80 (4.77)	18.89 (3.60)	35.84 (8.70)	32.46 (6.19)	45.69 (9.68)	39.78 (3.97)	7.79 (0.86)	8.60 (1.09)
MET	1.90 (0.27)	2.00 (0.33)	6.25 (1.37)	5.39 (1.03)	10.33 (2.54)	9.28 (1.78)	13.04 (2.78)	11.36 (1.14)	2.22 (0.25)	2.47 (0.30)
VE [l]	11.36 (3.59)	11.15 (2.81)	29.26 (10.35)	26.69 (10.35)	58.71 (21.96)	57.44 (19.81)	77.14 (25.76)	79.40 (26.03)	16.29 (5.10)	20.16 (6.32)
RQ	0.78 (0.07)	0.80 (0.04)	0.78 (0.06)	0.81 (0.06)	1.01 (0.11)	1.06 (0.12)	1.07 (0.11)	1.20 (0.09)	0.90 (0.07)	1.00 (0.17)
PetCO_2_ [mmHg]	30.25 (2.43)	30.63 (1.85)	36.50 (3.16)	35.38 (3.11)	39.00 (3.89)	38.50 (3.25)	38.50 (5.42)	36.00 (2.98)	28.43 (2.12)	27.24 (2.52)
VE/V̇O_2_	30.44 (3.03)	29.91 (2.55)	23.92 (2.34)	26.15 (4.21)	29.27 (3.21)	30.74 (4.8)	30.55 (5.37)	36.42* (5.02)	31.06 (2.45)	37.11 (7.75)
VE/V̇CO_2_	34.60 (2.97)	33.38 (2.67)	30.82 (1.53)	32.36 (1.53)	29.20 (1.78)	29.28 (1.76)	28.55 (1.76)	30.66 (1.76)	41.30 (1.72)	43.60 (1.72)

*p < 0.05 for the group effect.

**Figure 2:**
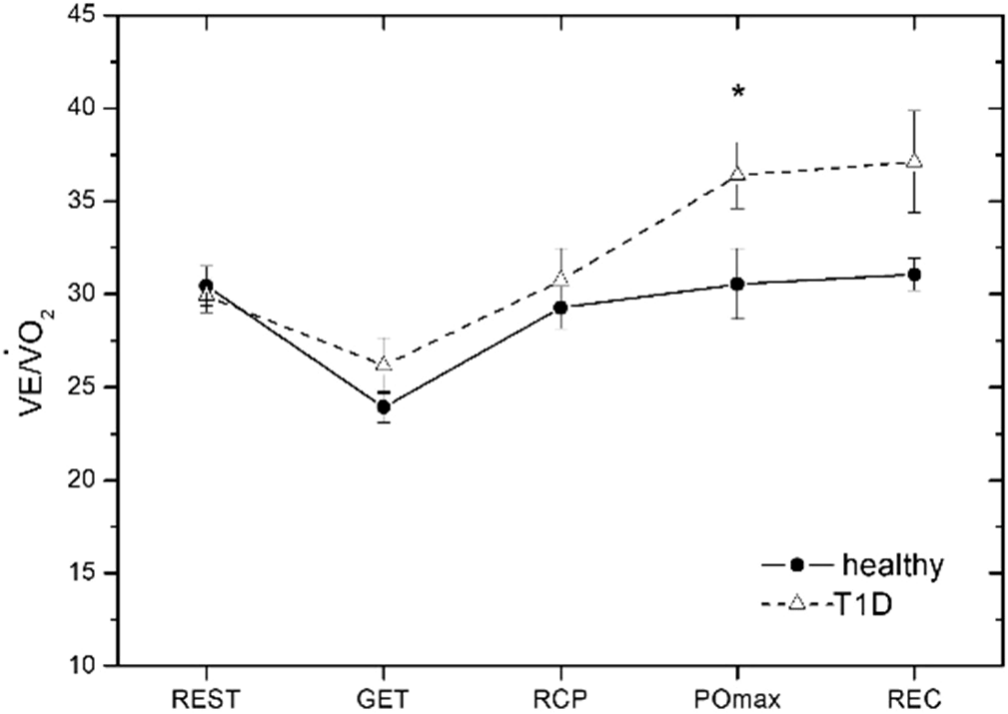
Time course of the ventilatory equivalent for oxygen (VE/V̇O_2_) throughout the measurement for both groups: the resting phase (REST), the gas exchange threshold (GET), the respiratory compensation point (RCP), the point of maximum power output (PO_max_) and the recovery phase (REC), *p < 0.01.

For the parameters HR, V̇O_2_, V̇O_2_/kg, MET, RQ, VE, VE/V̇CO_2_, PetCO2 and V̇O_2_/HR, a statistically significant main effect of time was observed (**Table 4**), while no significant group effect or interaction was found. For the ventilation equivalent for O2 (VE/V̇O_2_), both a statistically significant main effect of time (**Table 4**) and a significant group effect were observed (F (1, 14) = 4.732; p = 0.047, η^2^ = 0.253), while no interaction was found. In *post hoc* analysis, VE/V̇O_2_ at peak power showed a significant difference between groups (**Figure 2**).

**Table 4:** Results of the ANOVA test for effect of time for metabolic, respiratory system, and HR/HRV parameters.

Parameter	DF	F	p	η^2^
HR [min^-1^]	4	224.24	<0.001*	0.94
V̇O_2_ [ml]	4.56	109.18	<0.001*	0.74
V̇O_2_/kg [ml/kg]	4.56	247.46	<0.001*	0.90
MET	4.56	240.96	<0.001*	0.89
RQ	4.56	59.36	<0.001*	0.69
VE [l]	4.56	100.21	<0.001*	0.75
PetCO_2_ [mmHg]	4.56	67.26	<0.001*	0.66
VE/V̇O_2_	4	28.64	<0.001*	0.67
VE/V̇CO_2_	4	31.23	<0.001*	0.69
V̇O_2_/HR [ml/beat]	4.56	54.74	<0.001*	0.57
RR [ms]	1	36.67	<0.001*	0.72
SDNN [ml/kg]	1.14	16.78	0.001*	0.29
RMSSD	1.14	7.88	0.01*	0.16
LF [ms^2^]	1.14	14.60	0.002*	0.28
LF [n.u.]	1.14	9.03	0.01*	0.16
HF [ms^2^]	1.14	21.83	<0.001*	0.30
HF[n.u.]	1.14	6.29	0.03*	0.20
LF/HF	1.14	8.02	0.01*	0.21

DF, degrees of freedom; F, test statistic; p, p-value; η^2^ – effect size (explained variance); *p < 0.05.

**Table 5** shows the results of analysis for parameters, which were measured only once per exercise test. For the average slope of V̇O_2_/PO, healthy individuals achieved significantly higher values compared to individuals with T1D. No statistically significant difference between groups was observed for neither OUES or the VE/V̇CO_2_ slope (**Table 5**).

**Table 5:** t test statistic, degrees of freedom, p value and effect sizes of parameters, measured only once per participant.

Parameter	Healthy	T1D	95%CI	t(df)	p	Effect size
VE/V̇CO_2_ slope	27.91 (2.57)	30.54 (4.77)	-3.02/8.27	1.1 (14)	0.31	0.39
OUES	2689.38 (792.97)	2213.63 (639.72)	-379/1330	1.32 (14)	0.23	0.47
V̇O_2_/PO [ml/min/W]	9.99 (0.84)	8.45* (0.99)	0.23/2.85	2.77 (14)	0.028*	0.98
HRR30 [min^-1^]	23.08 (11.76)	20.17 (12.20)	-3.26/11.26	1.3 (14)	0.23	0.46
HRR60 [min^-1^]	39.94 (12.29)	36.52 (7.52)	-2.92/10.67	1.35 (14)	0.22	0.48

t-t statistics, df – degrees of freedom, effect size – Cohen’s d, *p < 0.05.

Results of the evaluation of cardiovascular response are presented in **Tables 3**–**7**. No statistically significant group or interaction effect was detected for parameters of cardiac function (HR, HRR30, HRR60, SDNN, RMSSD, LF, HF, LF/HF). However, a main effect of time was observed for heart rate and HRV parameters (**Table 4**).

**Table 6:** HRV parameters for both study groups during resting (REST) and recovery (REC) phase. Data are presented as mean values (standard deviation).

Parameter	REST		REC	
	Healthy	T1D	Healthy	T1D
RR [ms]	648.92 (129.28)	663.11 (113.90)	559.29 (80.51)	538.73 (51.28)
SDNN [ms]	54.88 (23.71)	57.68 (18.29)	29.21 (17.48)	29.44 (29.29)
RMSSD [ms]	34.89 (25.05)	34.00 (12.83)	18.51 (15.79)	17.72 (24.73)
LF [ms^2^]	1604.70 (1445.36)	1736.07 (1234.68)	523.35 (738.73)	342.36 (628.04)
LF [nu]	101.99 (38.14)	100.35 (27.31)	124.86 (45.58)	136.92 (30.48)
HF [ms^2^]	661.38 (727.39)	641.41 (416.26)	158.11 (255.52)	52.41 (97.86)
HF [nu]	40.05 (20.75)	38.50 (9.58)	30.19 (17.36)	20.59 (8.56)
LF/HF	3.91 (3.33)	2.89 (1.54)	7.53 (6.64)	8.77 (6.37)

**Table 7:** Mean values (standard deviation) for parameters associated with microcirculation in healthy participants and adolescents with T1D during resting (REST) and recovery (REC) phases.

Parameter	REST		REC	
	Healthy	T1D	Healthy	T1D
MAP [mmHg]	96.62 (20.79)	106.86 (21.78)	84.66 (10.73)	89.24 (20.77)
BF_fin_ [AU]	190.25 (78.96)	101.00 (39.18)	249.13 (91.76)	153.50 (66.33)
BF_arm_ [AU]	11.75 (3.45)	15.88 (6.15)	14.63 (6.74)	18.00 (5.88)
CVC_fin_ [AU/mmHg]	2.12 (1.10)	0.93 (0.42)	2.97 (0.87)	1.72 (0.57)
CVC_arm_ [AU/mmHg]	0.13 (0.04)	0.14 (0.05)	0.22 (0.10)	0.22 (0.09)
T_fin_ [°C]	31.95 (2.37)	29.68 (2.95)	34.48 (0.45)	33.17 (1.91)
T_arm_ [°C]	32.38 (1.44)	32.34 (0.56)	32.14 (1.35)	32.09 (1.09)

**Figure 3:**
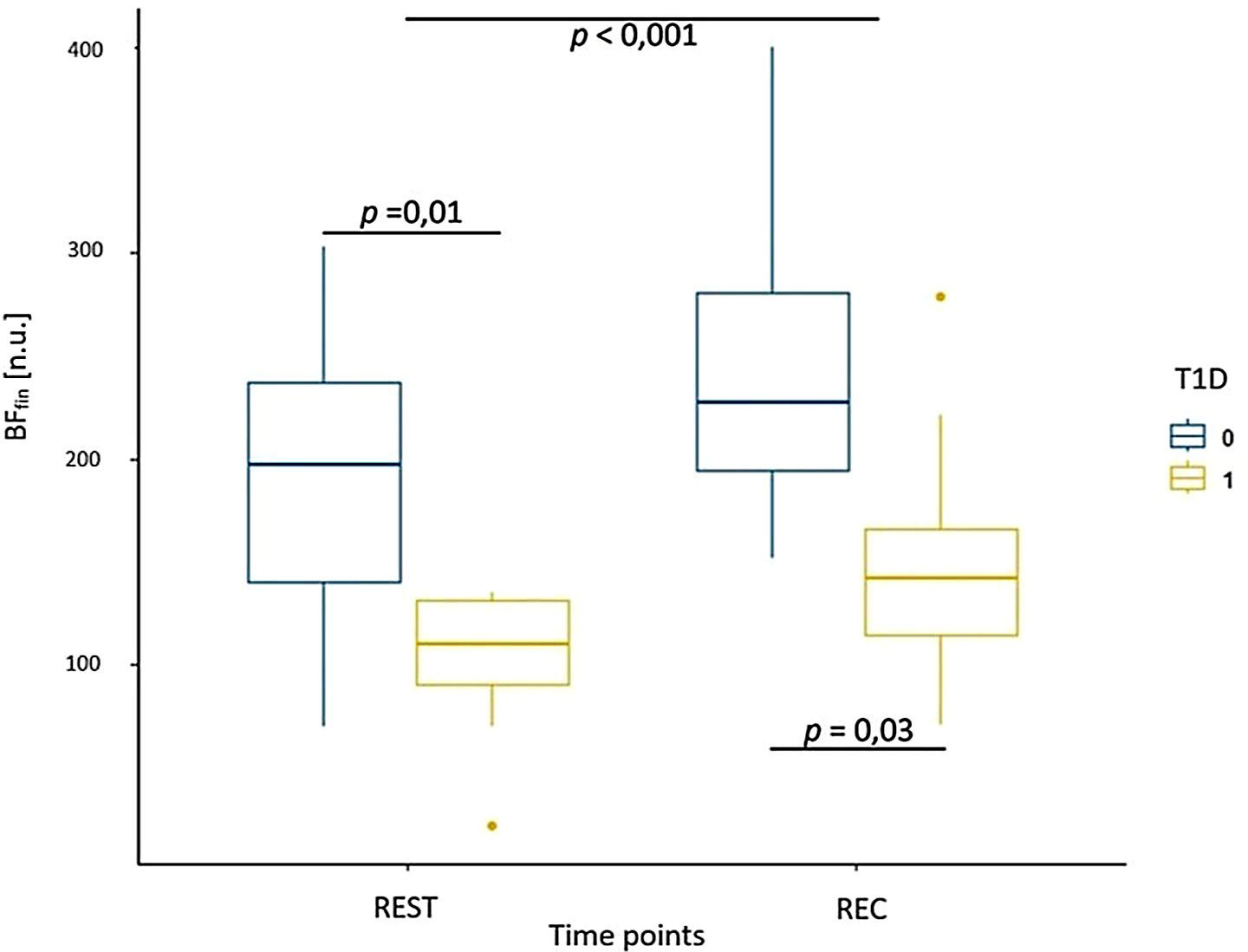
Box plot of skin blood flow measured in the finger during resting phase (REST) and during the recovery period (REC) for healthy participants (T1D 0) and adolescents with T1D (T1D 1).

**Figure 4:**
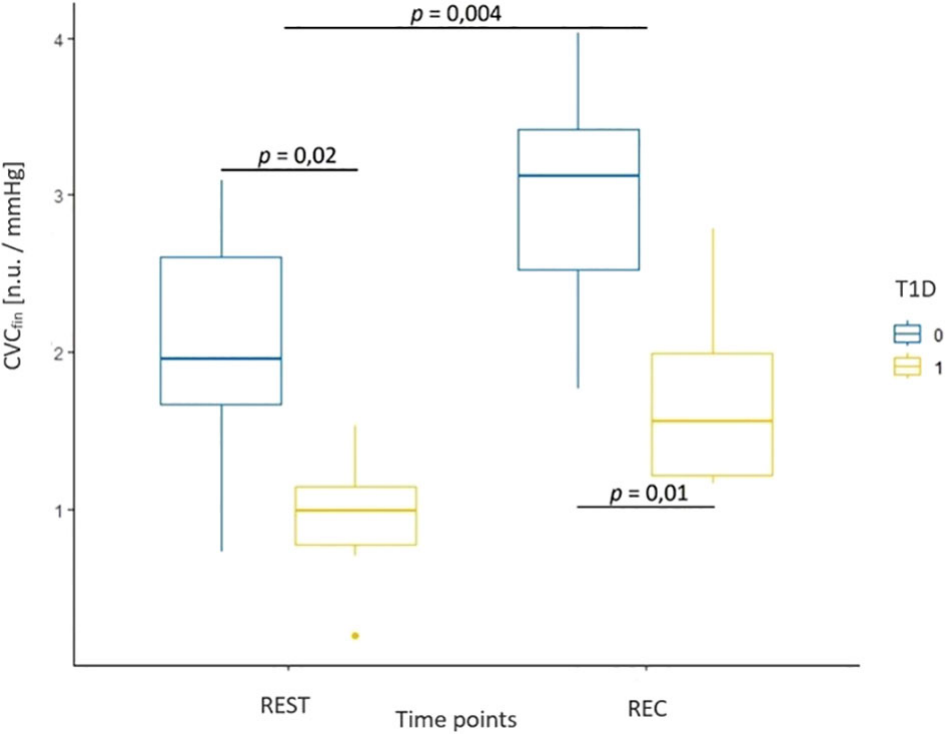
Box plot of cutaneous vascular conductance measured in the finger during resting phase (REST) and during the recovery period (REC) for healthy participants (T1D 0) and adolescents with T1D (T1D 1).

**Figure 5:**
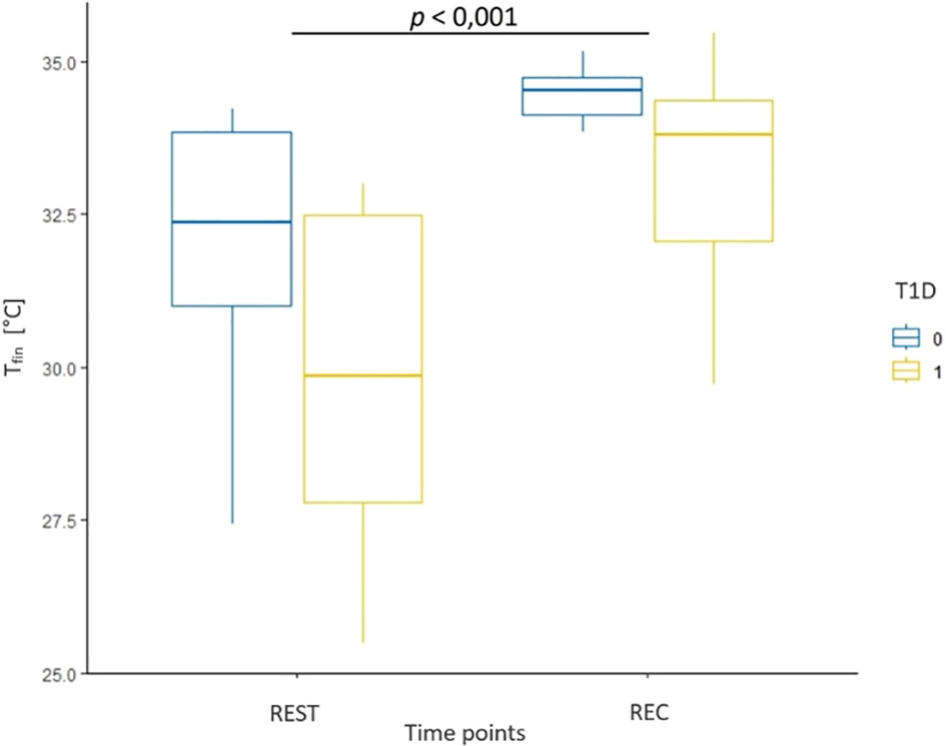
Box plot of skin temperature measured in the finger during resting phase (REST) and during the recovery period (REC) for healthy participants (T1D 0) and adolescents with T1D (T1D 1).

Results from the evaluation of skin microcirculation in both study groups is presented in **Table 7**. Regarding skin blood flow measured in finger pulp during resting and recovery periods, the results of the ANOVA test showed a statistically significant main effect of time (F (1, 14) = 17.22, p < 0.001, η² = 0.15) and group (F (1, 14) = 7.72, p = 0.01, η² = 0.32), but no significant interaction (**Figure 3**). In contrast, for skin blood flow in the forearm measured during the same periods, no statistically significant effects of time, group, or their interaction were detected. For cutaneous vascular conductance in the finger (CVC_fin_) measured during the resting and recovery period, the results showed both a significant effect of time (F (1, 14) = 14.41, p = 0.004, η² = 0.31) and a significant effect of group (F (1, 14) = 12.16, p = 0.006, η² = 0.46), while no interaction was observed (**Figure 4**). In contrast, only a significant effect of time was found (F (1, 14) = 9.47, p = 0.01, η² = 0.21) for CVC measured in the forearm (CVC_arm_), with no significant effect of group or interaction. The ANOVA results for finger temperature, measured during the resting and recovery periods, revealed a significant effect of time (F (1, 14) = 18.86, p < 0.001, η² = 0.36) and group (F(1, 14) = 4.87, p = 0.04, η² = 0.17), with no significant interaction (**Figure 5**). In contrast, the results for forearm temperature showed no statistically significant effects of time, group, or interaction.

Blood glucose and lactate concentrations at rest and peak power are presented in **Table 8**.

**Table 8:** Blood glucose (GC) and lactate concentrations (LC) measured during resting phase and upon reaching PO_max_, for both study groups. Data is presented as means (standard deviation).

Parameter	REST		POmax	
	Healthy	T1D	Healthy	T1D
GC [mmol/l]	5.56 (0.92)	11.34 (3.61)	5.55 (0.82)	10.40 (3.28)
LC [mmol/l]	2.96 (0.98)	2.25 (0.88)	8.98 (3.15)	10.33 (2.65)

**Figure 6:**
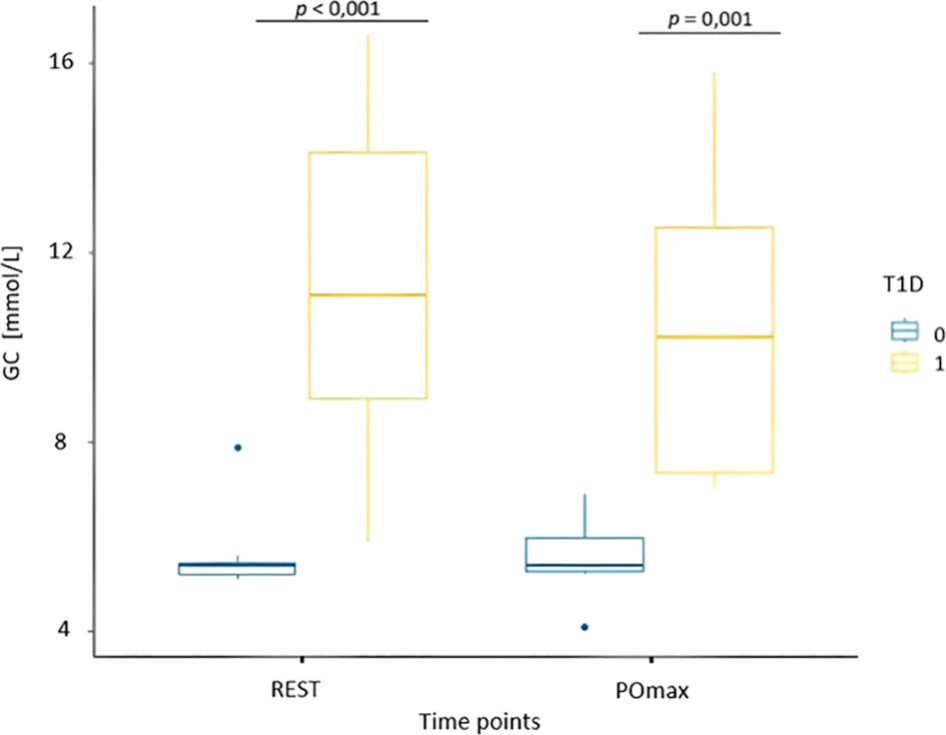
Box plot of blood glucose concentrations (GC) at the time of resting phase (REST) and immediately following PO_max_ for healthy participants (T1D 0) and adolescents with T1D (T1D 1).

**Figure 7:**
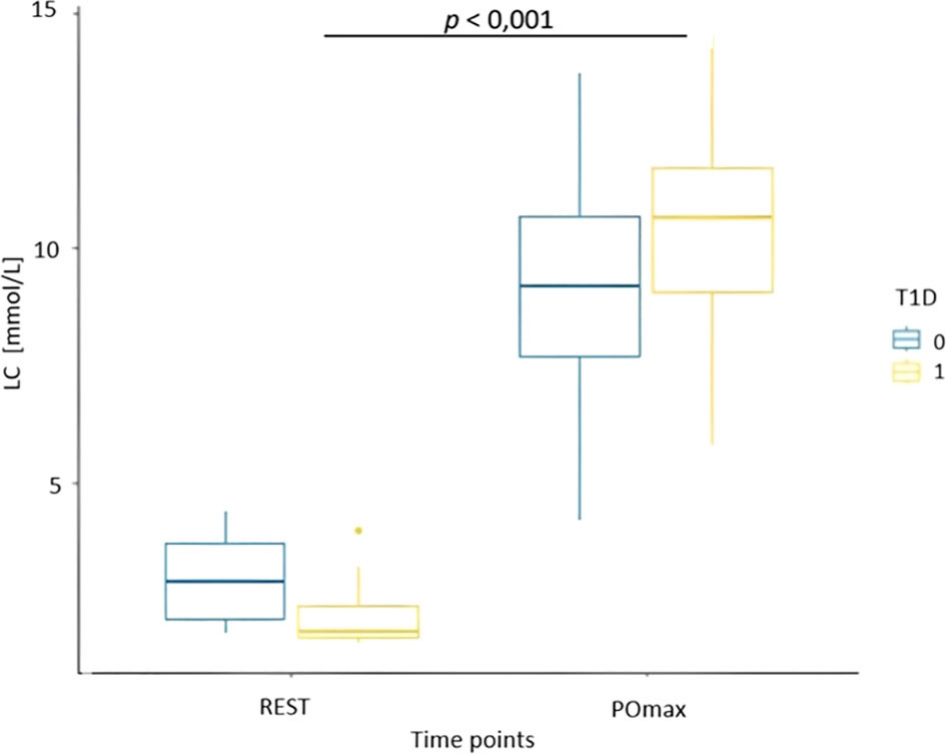
Box plot of blood lactate concentrations (LC) at the time of resting phase (REST) and immediately following PO_max_ for healthy participants (T1D 0) and adolescents with T1D (T1D 1).

The ANOVA results for blood glucose concentration showed a significant group effect (F(1, 14) = 18.26, p < 0.001, η² = 0.56), while no significant time or interaction were observed (**Figure 6**). In contrast, for blood lactate concentration, a significant time effect was found (F(1, 14) = 73.90, p < 0.001, η² = 0.75), with no significant group or interaction (**Figure 7**).

## Discussion

In this study, adolescents with T1D exhibited preserved overall exercise capacity and cardiovascular responses during exercise testing, demonstrated by comparable peak power output, V̇O_2max_, heart rate responses, and heart rate variability compared to healthy peers. However, subtle differences in oxygen utilization and peripheral vascular function were observed. Participants with T1D demonstrated a lower V̇O_2_/PO slope and a higher ventilatory equivalent for oxygen at maximal effort, suggesting reduced efficiency of oxygen uptake relative to work performed. In addition, skin blood flow and cutaneous vascular conductance in the finger pulp were significantly lower both at rest and during recovery. Together, these findings suggest that although central cardiovascular and autonomic responses to exercise appear largely preserved in adolescents with T1D, early changes in microvascular regulation and oxygen utilization may already be present.

The absence of significant circulatory or ventilatory constraints in adolescents with T1D is further supported by the absence of significant difference between groups in haemodynamic parameters and OUES, suggesting that peripheral metabolic and microvascular factors most likely contributed to the altered exercise response in T1D. The increase of the VE/V̇O_2_ ratio at peak exercise suggests an increased ventilatory cost relative to oxygen uptake, likely driven by a lower V̇O_2_ achieved for a given ventilatory response rather than impaired ventilatory efficiency, which, when present, manifested even at submaximal intensities (32). Additionally, no difference in VE/V̇CO_2_ between groups demonstrates the same ventilation-perfusion heterogeneity during exercise in individuals with T1D compared to healthy peers.

Our study found no significant difference for V̇O_2max_ between youth with T1D and healthy peers. Similar results were reported in studies on both adolescents, as well as young adults with T1D (36, 37). Conversely, several studies have indicated that both adolescents and adults with T1D attain lower peak V̇O_2_ values compared to healthy controls (13, 15, 21, 38), and that both the gas exchange threshold and the respiratory compensation point occur at lower V̇O_2_ levels in these individuals (13). Similarly variable outcomes have been reported regarding the effect of T1D on maximal power output achieved during exercise testing. A recent meta-analysis by De Visser et al. found that young individuals with T1D had lower V̇O_2max_ values and engaged in less physical activity compared to healthy peers, with differences becoming more pronounced when objective measures of physical activity were used (39). Habitual physical activity was comparable between our study groups, which could explain the similar V̇O_2max,_ achieved by individuals with T1D compared to their healthy peers.

In this study, we found no significant differences in parameters of cardiac function between healthy individuals and participants with T1D. This applied to all measured variables, including heart rate at various stages of exercise, heart rate recovery following exercise, and heart rate variability (HRV) both in resting and recovery periods. The lack of significant difference in heart rate and HRV is consistent with findings from some previous studies comparing healthy individuals and participants with T1D (22, 40), indicating preserved cardiovascular autonomic function. In contrast, published data suggests that HRV may already be attenuated in young adults with T1D, particularly during exercise-heat stress (41). A large study by Eckstein et al. in adults with T1D also showed significantly lower heart rates in individuals with T1D at maximal exertion, GET and RCP points, as well as lower heart rate reserve (13). Moser et al. also reported different heart rate patterns in young adults with T1D during exercise testing compared to healthy peers (36). Conversely, Turinese et al. found no significant difference in the cardiovascular response to CPET in young adults with T1D compared to individuals without T1D (15). Results on heart rate recovery values in our study group indicate a normal physiological response and do not suggest autonomic nervous system impairment in these individuals. Mean systolic arterial pressure also changed comparably in both groups over the course of the study, with higher values at rest prior to exercise and lower values during the recovery period. This is considered a normal response, as blood pressure decreases due to increased venous pooling of blood, while cardiac output declines more rapidly than systemic vascular resistance recovers (42).

The seemingly conflicting results from studies highlight the variability in cardiovascular autonomic responses among individuals with T1D and suggest that differences may depend on factors such as disease duration, glycaemic control, fitness level, and testing conditions. Our results support the notion that cardiac autonomic function may still be preserved in adolescents with T1D at rest and during recovery following graded exercise. It should be noted that both individuals with T1D as well as the control group in this study exhibited relatively high baseline heart rate values prior to CPET (93.25 and 95.12 min^-1^, respectively), which could reflect anticipatory sympathetic activation immediately prior to CPET (43), rather than a consequence of sedentary lifestyle or insufficient physical activity.

We observed significantly lower skin blood flow, measured at the fingertip, in individuals with T1D compared to healthy controls both during resting and recovery periods. However, in both study groups, a similar increase in blood flow was measured during the recovery period relative to the resting phase. In contrast, the response in forearm skin blood flow showed neither a significant group nor time effect. Similarly, Sorelli et al. reported reduced microvascular perfusion in adults with T1D at baseline, and a larger vasodilatory reserve after thermal stimulation, which is consistent with our observations (44). In contrast, Pichler et al. reported a significantly lower forearm blood flow at rest, along with significantly smaller increases in blood flow in this area following exercise in children and adolescents with T1D, compared with healthy peers (45). Evidence of microvascular dysfunction in adolescents and young adults as measured by forearm perfusion following local heating was also reported by Shah et al. (46). In a study on adults with T1D, Saloň et al. reported no retinal microvascular changes during or following CPET, highlighting the need for further research regarding the effect of T1D on microvascular response to exercise testing (23).

We found similar results for cutaneous vascular conductance (CVC). CVC measured at the fingertip was significantly reduced in individuals with T1D compared to healthy controls, and both groups experienced a significant yet similar increase in CVC during the recovery period relative to the resting phase. In the forearm, only a time effect was observed, with CVC increasing during recovery. Similarly, skin temperature showed the greatest increase in the finger following exertion, while the skin temperature of the forearm did not show any significant differences. These consistent changes across parameters are physiologically plausible, as blood flow depends on CVC, which in turn is influenced by local tissue temperature. These results are similar to those obtained in a study on healthy young males, supporting the hypothesis that glabrous skin areas might overtake the thermoregulatory role in the recovery phase (47).

These results might suggest that early-stage microvascular imparment or endothelial dysfunction in glabrous skin may already be present in individuals with T1D, demonstrated by the reduced baseline blood flow measured in the fingertip, while the acute vasodilatory response in this group remained largely preserved, indicating a relatively intact microvascular autoregulatory capacity.

Glabrous skin is characterized by dense sympathetic innervation and abundant arteriovenous anastomoses, rendering its perfusion highly sensitive to autonomic and endothelial influences. As such, glabrous skin blood flow may serve as a sensitive indicator of early microvascular dysfunction in T1D, complementing traditional measures of cardiopulmonary exercise performance. Additionally, its unique characteristics allows glabrous skin to play a central role in thermoregulation, allowing rapid adjustments in skin blood flow to facilitate heat dissipation. The markedly lower skin blood flow and cutaneous vascular conductance observed in individuals with T1D therefore suggest not only altered microvascular regulation but also a potential impairment in thermoregulatory capacity, which has also been reported in other studies (48).

When assessing the microcirculatory response in people with diabetes, the effect of insulin on cutaneous blood flow should be considered. Insulin has important vascular actions and, under physiological conditions, generally promotes vasodilation via endothelial nitric oxide signaling. However, in the presence of endothelial dysfunction or altered vascular control, this vasodilatory effect of insulin may be attenuated, and the balance may shift toward relative vasoconstriction (49). This mechanism is particularly relevant for microvascular blood flow in non-glabrous skin such as the forearm. In our study, however, differences in skin blood flow were observed only at the fingertip, that is, in glabrous skin, where thermoregulatory and sympathetic vascular regulation are likely to play a more important role than endothelial function alone and the effect of insulin might be indirect. However, since insulin dosing was individual and not recorded as a study variable, its contribution to this site-specific response cannot be determined. Further research is needed to determine the effect of exogenous insulin administration on microcirculation in this setting.

At both the beginning and following CPET, individuals with T1D had higher blood glucose concentrations compared to healthy participants. Given the nature of T1D, elevated blood glucose levels are entirely expected and align with results of similar studies (15). Several other studies have reported no significant change in blood glucose concentrations from the beginning to the end of exercise testing in both healthy individuals and people with T1D (14, 15). However, the lack of data regarding insuling dosing in individuals with T1D limits interpretation of glucose dynamics during exercise testing in this study.

Before and after exercise, lactate values did not differ significantly between the two groups. However, the mean post-exercise lactate concentrations indicate that participants reached, or closely approached, maximal exertion.

Some limitations of this study should be considered. First, the relatively small sample size is an important limitation of this study. Although the cohort met the minimum size estimated by *a priori* power analysis for the primary analysis, a larger sample would have increased statistical power and improved external validity. Therefore, the findings should be interpreted cautiously as preliminary evidence that requires confirmation in larger and more representative cohorts. Second, glucose levels were assessed only at the beginning and end of exercise, limiting insight into glycemic dynamics during exertion. Furthermore, hydration status was not assessed in either group. This may have influenced autonomic measures, particularly HRV-derived parasympathetic indices (50), but is less likely to have affected the cutaneous microvascular measurements obtained under standardized local testing conditions (51). Due to the short duration of the exercise protocol and in line with current consensus recommendations (52), no standardized adjustments to personalised insulin dosing of each participant with T1D were made. Similarly, while participants were advised to have a meal at least one hour before the exercise protocol, meals were not standardized and information regarding the timing and content of meals were not systematically collected. Additionally, pre-test continuous glucose monitoring trends, insulin dosing data and blood ketone levels were not systematically analysed in the T1D group, since this data was not relevant for the healthy control group and could not be collected in a comparable manner across both groups. However, these factors may influence physiological responses to exercise and should be evaluated as within-subject determinants of CPET responses in larger cohorts.

## Conclusion

Our study has shown no significant differences in peak power output, maximum oxygen uptake or cardiovascular parameters such as heart rate, heart rate variability and heart rate recovery, suggesting that adolescents with T1D may have preserved cardiovascular autonomic function and comparable aerobic capacity to their healthy peers. However, significant differences in oxygen consumption per power output and in the ventilatory equivalent for oxygen indicate a possible subclinical inefficiency in oxygen utilization during high-intensity exercise in the T1D group. Additionally, the attenuated response in skin blood flow and cutaneous vascular conductance in the glabrous skin of T1D study participants suggest early signs of microvascular dysfunction, emphasizing the need for ongoing monitoring of vascular function even in young diabetic individuals without evident complications. Further research is warranted to provide insight in the exact mechanism of microvascular dysfunction in T1D, potentially using specific inhibitors of vascular tone mediators.

These findings support the hypothesis that early in disease progression, type 1 diabetic adolescents may already exhibit differences in metabolic, respiratory, and skin microvascular response to short-term dynamic exercise. There is a need for future studies on larger cohorts to elucidate the relationship between indicators of glycemic control, as well as glycemic levels throughout exercise testing, with various physiological adaptations to exercise, particularly the function of microcirculation. Longitudinal research is warranted to understand how these parameters evolve over time and influence long-term health outcomes.

## Data Availability

The raw data supporting the conclusions of this article will be made available by the authors, without undue reservation.
